# WHO ENJOYS THE GOLDEN YEARS? RACIAL AND SOCIOECONOMIC DISPARITIES IN YEARS OF HEALTHY RETIREMENT

**DOI:** 10.1093/geroni/igae098.2142

**Published:** 2024-12-31

**Authors:** Leah Abrams, Yuan Zhang

**Affiliations:** Tufts University, Medford, Massachusetts, United States; Columbia University, New York, New York, United States

## Abstract

For several decades, until around 2010, Americans enjoyed increases in life expectancy, and for some, extended disability-free life expectancy. However, mortality progress has since stagnated, Americans with low education have not benefited from delayed disability, and there remain substantial racial disparities in morbidity and mortality. We aim to determine who in American society retains health and functioning to enjoy retirement years by simultaneously examining healthy life expectancy and time spent in retirement. We use data on physical functioning and work status from the Health and Retirement study in two periods (1998 and 2016) to run Markov models that calculate the number of years and percent of remaining life in healthy work, unhealthy work, healthy retirement, and unhealthy retirement by race and socioeconomic status. Results to date reveal that, in 2016 compared to 1998, adults over age 51 spent a smaller portion of their life in healthy retirement, with a shift toward healthy work. In 2016, Black and white men and women all spent between 17-20% of remaining life years after 51 in healthy retirement, but they arrived there differently. Men spent about 42% of life years healthy and working, compared to about 30% for women. Women spent more time not working with functional limitations. Looking to race, white men spent a greater portion of time working with functional limitations, whereas Black men were not working with functional limitations. Black women spent more time working with functional limitations and less time not working, with and without functioning.

